# Diphenyl Diselenide Alleviates Tert-Butyl Hydrogen Peroxide-Induced Oxidative Stress and Lipopolysaccharide-Induced Inflammation in Rat Glomerular Mesangial Cells

**DOI:** 10.3390/ijms231911215

**Published:** 2022-09-23

**Authors:** Xing Wang, Yi Huan, Shuainan Liu, Caina Li, Hui Cao, Lei Lei, Quan Liu, Wenming Ji, Sujuan Sun, Kaixun Huang, Jun Zhou, Zhufang Shen

**Affiliations:** 1 School of Pharmacy, North Sichuan Medical College, Nanchong 637100, China; 2 State Key Laboratory of Bioactive Substances and Functions of Natural Medicines, Key Laboratory of Polymorphic Drugs of Beijing, Institute of Materia Medica, Chinese Academy of Medical Sciences and Peking Union Medical College, Beijing 100050, China; 3 Hubei Key Laboratory of Bioinorganic Chemistry & Materia Medica, Hubei Engineering Research Center for Biomaterials and Medical Protective Materials, Key Laboratory of Material Chemistry for Energy Conversion and Storage, Ministry of Education, School of Chemistry and Chemical Engineering, Huazhong University of Science and Technology, 1037 Luoyu Road, Wuhan 430074, China; 4 Shenzhen Huazhong University of Science and Technology Research Institute, Shenzhen 518057, China

**Keywords:** diphenyl diselenide, selenium, diabetic nephropathy, oxidative stress, inflammation

## Abstract

Hyperglycemia, oxidative stress, and inflammation play key roles in the onset and development of diabetic complications such as diabetic nephropathy (DN). Diphenyl diselenide (DPDS) is a stable and simple organic selenium compound with anti-hyperglycemic, anti-inflammatory, and anti-oxidative activities. Nevertheless, in vitro, the role and molecular mechanism of DPDS on DN remains unknown. Therefore, we investigated the effects of DPDS on tert-butyl hydrogen peroxide (t-BHP)-induced oxidative stress and lipopolysaccharide (LPS)-induced inflammation in rat glomerular mesangial (HBZY-1) cells and explored the underlying mechanisms. DPDS attenuated t-BHP-induced cytotoxicity, concurrent with decreased intracellular ROS and MDA contents and increased SOD activity and GSH content. Moreover, DPDS augmented the protein and mRNA expression of Nrf2, HO-1, NQO1, and GCLC in t-BHP-stimulated HBZY-1 cells. In addition, DPDS suppressed LPS-induced elevations of intracellular content and mRNA expression of interleukin (IL)-6, IL-1β and TNF-α. Furthermore, LPS-induced NFκB activation and high phosphorylation of JNK and ERK1/2 were markedly suppressed by DPDS in HBZY-1 cells. In summary, these data demonstrated that DPDS improves t-BHP-induced oxidative stress by activating the Nrf2/Keap1 pathway, and also improves LPS-induced inflammation via inhibition of the NFκB/MAPK pathways in HBZY-1 cells, suggesting that DPDS has the potential to be developed as a candidate for the prevention and treatment of DN.

## 1. Introduction

One of the common and major microvascular complications of diabetes mellitus (DM) is diabetic nephropathy (DN), which is also the main cause of end-stage renal disease [1]. Results of an epidemiological survey have shown that about 40% of type 2 diabetic patients eventually develop DN. More seriously, patients with type 1 diabetes are more likely to develop DN [2]. DN is a progressive disease in which the main pathological changes of the kidney are the basement membrane and glomerular membrane thickening and glomerular hypertrophy, as well as excessive accumulation of extracellular matrix (ECM) protein [3]. The pathogenesis of DN is multiplex and complex, and, at the present time, is not fully understood. It is currently believed that mitogen-activated protein kinase (MAPK) signaling pathways, oxidative stress, lipid disorders, inflammatory pathways, polyol activation, and renal hemodynamic changes all contribute to the pathophysiology of DN [4]. In addition, although there are many therapeutic drugs for diabetic nephropathy, the number of patients with DN is still increasing due to the side effects and poor efficacy. Therefore, there is an urgent need to develop novel drugs that can more effectively prevent DN.

Glomerular mesangial cells, together with the ECM secreted by them, constitute the glomerular mesangial region, which is also a major functional cell in the glomerulus [5]. Mesangial cells play an important role in protecting the integrity of the renal tubular vascular bed, maintaining the balance of mesangial matrix and regulating glomerular filtration. Mesangial cells can promote cell proliferation and ECM deposition by secreting a variety of bioactive molecules and automatically activating them. They can also influence the function of glomeruli and other inherent cells through paracrine mechanism, thus participating in the process of glomerular injury and repair, and finally promoting the occurrence and development of glomerulosclerosis [6]. Therefore, studying the regulatory mechanism of drug candidates on the dysfunction of diabetic mesangial cells will provide a reliable theoretical basis for understanding and developing better drugs and therapeutic methods for DN.

Oxidative stress provoked by chronic hyperglycemia is a primary factor in the occurrence and development of DN [1]. Continuous hyperglycemia promotes the generation of reactive oxygen species (ROS) and free radicals, which subsequently activate various redox-responsive signal molecules, resulting in cell dysfunction and damage and eventually microvascular and macrovascular complications, such as DN [7]. Subsequently, oxidative stress (OS) provokes the activation of multiple intracellular signaling pathways and transcription factor expression [8], leading to elevated ECM deposition and decreased matrix degradation, bringing about glomerular dysfunction and renal fibrosis, and ultimately promoting the occurrence and development of DN [9]. In addition to OS, inflammation is also involved in the progression of DN [10,11]. Various studies have shown that there are a large number of inflammatory cells in the kidney of DN animal models and patients [12]. The accumulation of inflammatory cells in the kidney causes certain damage to the glomeruli and renal tubules, thus enhancing DN progression [13]. Therefore, compounds with anti-inflammatory and antioxidant activities may be able to improve DN.

**Figure 1:**
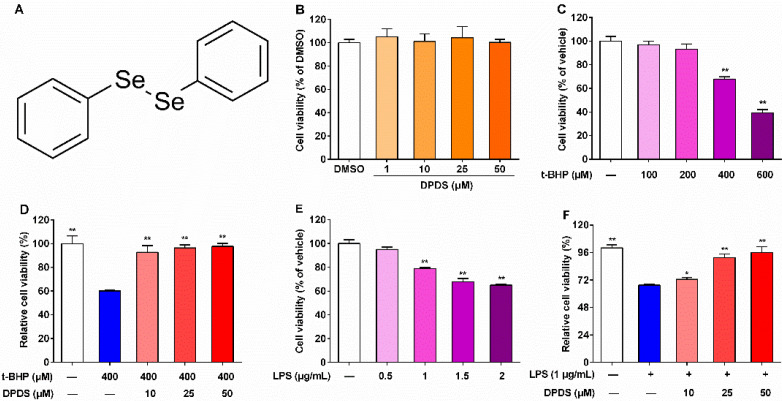
DPDS protected HBZY-1 cells against t-BHP-induced injury. The HBZY-1 cells were exposed to indicated concentrations of agents for 24 h, and cell viability was detected by a CCK-8 kit. (**A**) Chemical structure of DPDS. (**B**) Cell viability of HBZY-1 cells after treatment with indicated concentrations of DPDS for 24 h (*n* = 3). Reference: DMSO group. (**C**) t-BHP-induced cytotoxicity in HBZY-1 cells (*n* = 3). Reference: vehicle group. (**D**) Effects of DPDS on t-BHP-induced cytotoxicity (*n* = 6). Reference: the 400 μM t-BHP group. (**E**) LPS-induced cytotoxicity in HBZY-1 cells (*n* = 6). Reference: vehicle group. (**F**) Effects of DPDS on LPS-induced cytotoxicity (*n* = 6). Reference: the 1 μg/mL LPS group. Data are presented as means ± SEM. * *p* < 0.05, ** *p* < 0.01.

Organoselenium compounds have attracted widespread interest for a long time due to their own low toxicity and potent pharmacological activities [14]. In this regard, diselenides are good antioxidant candidates due to their similar chemical characteristics to ebselen, a widely accepted antioxidant agent that mimics glutathione peroxidase (GPx) activity. In particular, diphenyl diselenide (DPDS, Figure 1A), a simple synthetic diaryl diselenide, has been reported to exhibit multiple beneficial bioactivities in some toxicity and disease models induced by chemical agents [15,16,17]. Intriguingly, it has been reported that DPDS possesses stronger antioxidant activity as a GPx mimic [18,19,20], stronger anti-inflammatory activity [19], and lower toxicity to rodents than ebselen [21,22]. Moreover, administration with DPDS effectively ameliorated the symptoms of hyperglycemia and inhibited hepatic oxidative stress in rats with streptozotocin (STZ)-induced diabetes [23]. More importantly, our previous study showed that DPDS supplementation alleviated nephropathy by regulating oxidative stress and inflammatory response in STZ-induced diabetic rats [24]. Nevertheless, the specific renal cell type on which DPDS acts remains unclear, given that there are multiple types of renal cells. In this regard, tert-butyl hydrogen peroxide (t-BHP)-induced oxidative stress and lipopolysaccharide (LPS)-induced inflammation in rat glomerular mesangial (HBZY-1) cells are often used as cellular models for DN research. However, to our knowledge, whether DPDS can protect glomerular mesangial cells from t-BHP-induced oxidative stress and LPS-induced inflammation, and the detailed mechanisms, have not been described. Therefore, our study aimed to evaluate the effect of DPDS on DN in two in vitro cell models and explore its possible molecular mechanism. Here, we found that DPDS attenuated t-BHP-induced oxidative stress by activating the Nrf2/Keap1 pathway, and alleviated LPS-induced inflammation via inhibiting the MAPK/NFκB pathways in HBZY-1 cells. Our research provides a new direction for the development of new organoselenium drugs for the prevention and treatment of DN, and provides an experimental basis for further exploration of the possibility of DPDS as a preventive and therapeutic drug for DN and experimental development.

## 2. Results

### 2.1. DPDS Protected HBZY-1 Cells against t-BHP and LPS-Induced Injury

Firstly, the cytotoxicity of DPDS on HBZY-1 cells was evaluated. DPDS at the tested concentrations (1–50 μM) had no significant effects on the viability of HBZY-1 cells (Figure 1B), suggesting the absence of cytotoxicity. Compared with the vehicle group, the t-BHP treatment (100–600 μM) dose-dependently reduced cell viability and, in particular, treatment with 400 and 600 μM t-BHP caused a statistically significant decrease (Figure 1C). However, treatment with DPDS at the tested concentrations dose-dependently increased cell viability in t-BHP-stimulated HBZY-1 cells (Figure 1D). Compared to the vehicle group, the LPS treatment (1–2 μg/mL) dose-dependently reduced cell viability (Figure 1E). However, treatment with DPDS at the tested concentrations dose-dependently increased cell viability in LPS-stimulated HBZY-1 cells (Figure 1F). Therefore, DPDS at the concentrations of 10, 25 and 50 μM protected HBZY-1 cells against t-BHP-induced injury.

### 2.2. DPDS Attenuated t-BHP-Induced Oxidative Stress in HBZY-1 Cells

**Figure 2:**
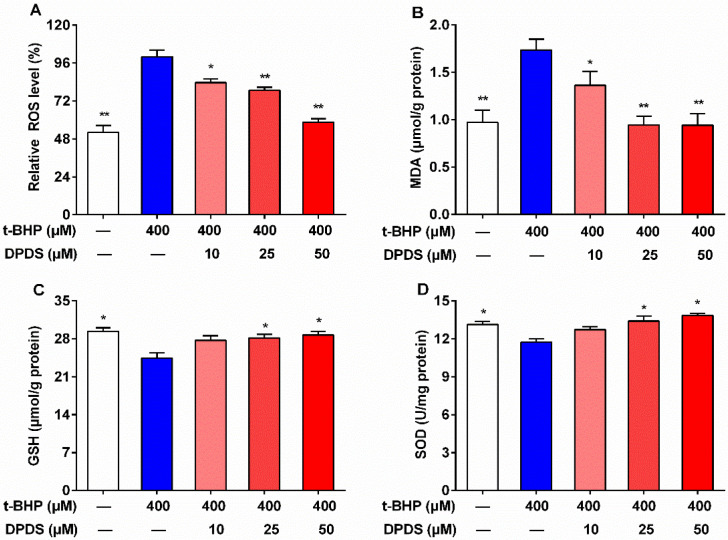
DPDS attenuated t-BHP-induced oxidative stress in HBZY-1 cells. The cells were incubated with t-BHP (400 μM) alone or in combination with various concentrations of DPDS for 24 h. The levels of ROS (**A**), MDA (**B**), and GSH (**C**) and SOD activity (**D**) were measured. Values are presented as means ± SEM, *n* = 4. * *p* < 0.05, ** *p* < 0.01 versus the 400 μM t-BHP group.

In order to ascertain whether the antioxidant action of DPDS is involved in its protective effects on t-BHP-induced cytotoxicity, reactive oxygen species and antioxidant factors in HBZY-1 cells were assessed. As anticipated, relative to the vehicle group, the t-BHP treatment significantly augmented ROS and MDA levels, and reduced GSH level and SOD activity (Figure 2A–D), suggesting the presence of t-BHP-induced oxidative stress. However, levels of ROS and MDA in t-BHP-stimulated HBZY-1 cells were significantly decreased after simultaneous treatment with DPDS at 10, 25 and 50 μM (Figure 2A,B). Meanwhile, after treatment with DPDS (25, 50 μM), intracellular GSH content and SOD activity were significantly augmented in t-BHP-stimulated HBZY-1 cells (Figure 2C,D). This indicates that DPDS plays a positive antioxidant role in t-BHP-stimulated HBZY-1 cells.

### 2.3. DPDS Activated the Nrf2/Keap1 Signaling Pathway in t-BHP-Stimulated HBZY-1 Cells

**Figure 3:**
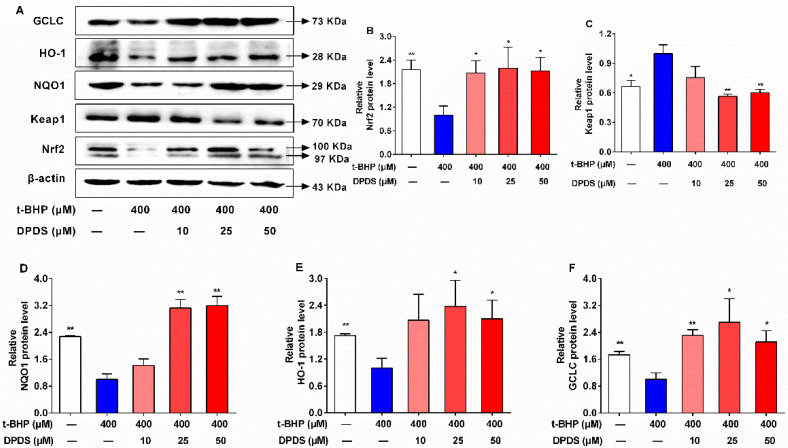
DPDS activated the Nrf2/Keap1 signaling pathway in t-BHP-stimulated HBZY-1 cells. HBZY-1 cells were exposed to t-BHP (400 μM) alone or in combination with DPDS (10, 25 or 50 μM) for 24 h. (**A**) Representative protein band of Nrf2, NQO1, HO-1 and GCLC. (**B**) The relevant quantitative data of Nrf2 expressed as fold of t-BHP group. (**C**) The relevant quantitative data of Keap1 expressed as fold of t-BHP group. (**D**) The relevant quantitative data of NQO1 expressed as fold of t-BHP group. (**E**) The relevant quantitative data of HO-1 expressed as fold of t-BHP group. (**F**) The relevant quantitative data of GCLC expressed as fold of t-BHP group. Values are presented as means ± SEM, *n* = 4. * *p* < 0.05, ** *p* < 0.01 versus the 400 μM t-BHP group.

Nrf2 is an important transcriptional activator of antioxidant genes and plays a key role in antioxidant protection [25]. To address the antioxidant mechanism of DPDS, its effects on the protein expression of Nrf2 and the downstream antioxidant enzymes were investigated by western blot [26]. Compared to the vehicle group, the t-BHP treatment led to significant decreases in the protein levels of Nrf2 and related antioxidant enzymes HO-1, NQO1, and GCLC, and resulted in an increase in the protein levels of Keap1 (Figure 3A–F). However, administration of DPDS for 24 h significantly promoted the protein levels of Nrf2 in t-BHP-stimulated HBZY-1 cells (Figure 3A,B). Moreover, treatment with DPDS increased the protein expression of Nrf2-related target proteins in t-BHP-treated HZBY-1 cells, including NQO1, HO-1, and GCLC (Figure 3A,D–F), and reduced the protein expression of Keap1 (Figure 3A,C). Altogether, the activation of the Nrf2/Keap1 pathway is connected to the antioxidant mechanism of DPDS in t-BHP-stimulated HBZY-1 cells.

### 2.4. DPDS Enhanced the Gene Expression of Nrf2 and Antioxidant Enzymes in t-BHP-Stimulated HBZY-1 Cells

**Figure 4:**
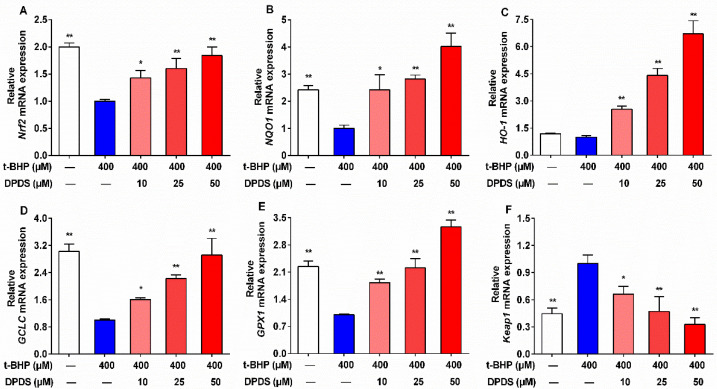
DPDS enhanced the gene expression of Nrf2 and antioxidant enzymes in t-BHP-stimulated HBZY-1 cells. HBZY-1 cells were incubated with t-BHP (400 μM) alone or in combination with indicated concentrations of DPDS for 24 h. The relative mRNA levels of *Nrf2* (**A**), *NQO1* (**B**), *HO-1* (**C**), *GCLC* (**D**), *GPX1* (**E**) and *Keap1* (**F**) expressed as fold of the t-BHP group. Gene levels were normalized against those of β-actin. Values are presented as means ± SEM, *n* = 3–4. * *p* < 0.05, ** *p* < 0.01 versus the 400 μM t-BHP group.

In order to further ascertain whether the improvement of OS by DPDS in t-BHP-stimulated HBZY-1 cells is connected to the Nrf2/Keap1 system, the relevant mRNA levels were analyzed by RT-qPCR. In the t-BHP group, the mRNA expression of *Nrf2*, *GCLC*, *NQO1*, and *GPX1* was downregulated, while the *Keap1* mRNA was upregulated in comparison with the vehicle group (Figure 4A,B,D–F). In addition, relative to the vehicle group, the gene expression of *HO-1* in the t-BHP group showed a decreasing trend, but there was no significant difference (Figure 4C). However, after treatment with DPDS for 24 h, the gene expression of *Nrf2*, *NQO1*, *HO-1*, *GCLC*, and *GPX1* was significantly upregulated, whereas the gene levels of *Keap1* were dose-dependently reduced in t-BHP-stimulated HBZY-1 cells (Figure 4), which further confirmed that DPDS could activate the Nrf2/Keap1 pathway in t-BHP-stimulated HBZY-1 cells.

### 2.5. DPDS Decreased Intracellular Contents and Gene Expression of Pro-Inflammatory Cytokines in LPS-Stimulated HBZY-1 Cells

**Figure 5:**
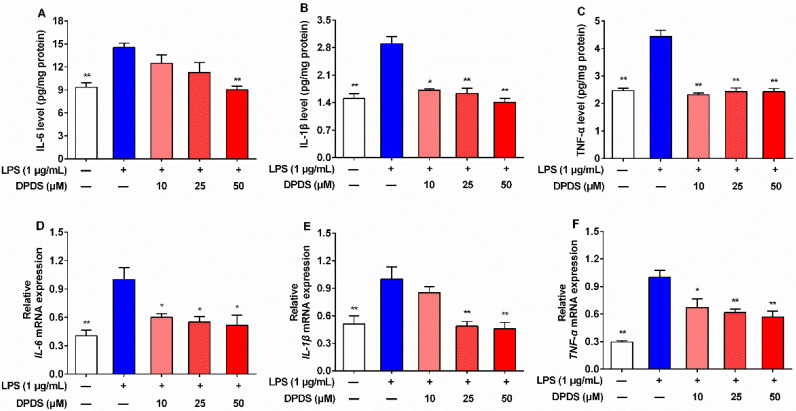
DPDS decreased intracellular contents and gene expression of inflammatory cytokines in LPS-treated HBZY-1 cells. HZBY-1 cells were incubated with LPS (1 μg/mL) alone or in combination with indicated concentrations of DPDS for 24 h. (**A**) Intracellular IL-6 content. (**B**) Intracellular IL-1β content. (**C**) Intracellular TNF-α content. The relative mRNA levels of *IL-6* (**D**), *IL-1β* (**E**) and *TNF-α* (**F**) expressed as fold of the LPS group. Values are presented as means ± SEM, *n* = 4. * *p* < 0.05, ** *p* < 0.01 versus the LPS group.

Since inflammation plays critical roles in the development of DN, the contents of pro-inflammatory cytokines in LPS-treated HBZY-1 cells were measured using ELISA kits. In comparison with the vehicle group, the LPS group showed significantly elevated intracellular IL-6, IL-1β, and TNF-α levels (Figure 5A–C), indicating the successful establishment of the inflammation model. However, after exposure to DPDS (10, 25 or 50 μM) for 24 h, the contents of these pro-inflammatory cytokines were significantly reduced (Figure 5A–C). Furthermore, relative to the vehicle group, the LPS-treated HBZY-1 cells exhibited markedly increased gene levels of *TNF-α*, *IL-6*, and *IL-1β* (Figure 5D–F), while administration of DPDS for 24 h effectively reversed these changes (Figure 5D–F). Collectively, DPDS obviously inhibited the LPS-induced inflammatory response in HBZY-1 cells.

### 2.6. DPDS Suppressed LPS-Induced NFκB and MAPK Signaling in HBZY-1 Cells

**Figure 6:**
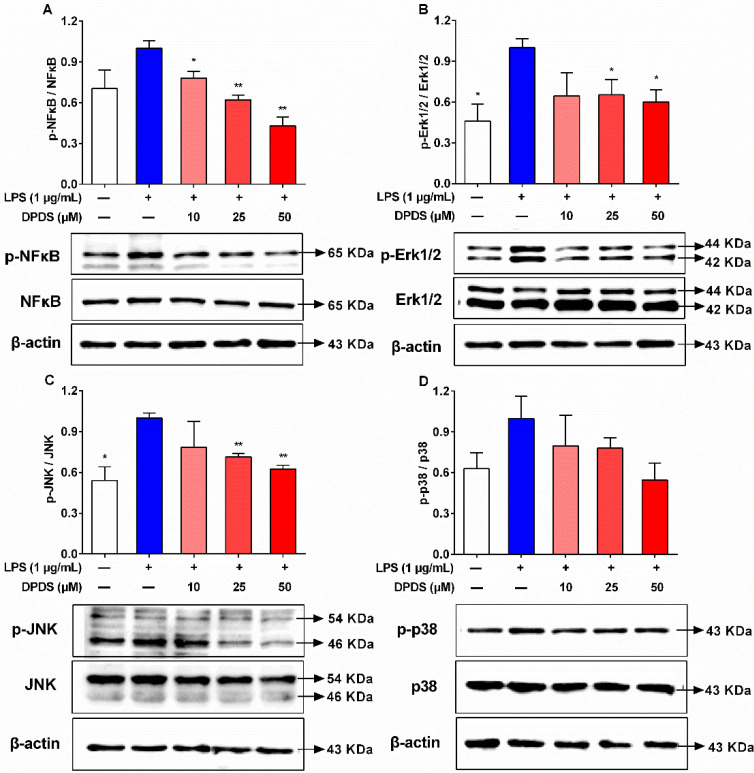
DPDS suppressed LPS-induced NFκB and MAPK signaling in HBZY-1 cells. HZBY-1 cells were exposed to LPS (1 μg/mL) alone or in combination with DPDS (10, 25 or 50 μM) for 24 h. (**A**) The protein bands of p-NFκB, NFκB and the ratios of p-NFκB/NFκB. (**B**) The protein bands of p-ERK1/2/, ERK1/2 and the ratios of p-ERK1/2/ERK1/2. (**C**) The protein bands of p-JNK, JNK and the ratios of p-JNK/JNK. (**D**) The protein bands of p-p38, p38 and the ratios of p-p38/p38. Values are presented as fold of the LPS group and as means ± SEM, *n* = 3. * *p* < 0.05, ** *p* < 0.01 versus the LPS group.

It is widely recognized that the NFκB signaling pathway is a classical pathway to modulate the immune response and inflammation. In addition, the MAPK family members (p38, JNK, and ERK1/2) are known to be involved in the expression of various inflammatory cytokines via activating NFκB [27,28]. To further address the mechanism underlying the protective effects of DPDS on LPS-induced inflammatory response, the protein levels and phosphorylation levels of NFκB were detected. The phosphorylation level of NFκB was dramatically increased after LPS stimulation in HBZY-1 cells (Figure 6A). However, the DPDS treatment significantly attenuated the phosphorylation level of NFκB in LPS-treated cells (Figure 6A). Next, we measured the total protein and phosphorylated protein levels of JNK, ERK1/2, and p38 in HBZY-1 cells. Compared with the vehicle group, the LPS-treated HBZY-1 cells exhibited significantly increased phosphorylation levels of ERK1/2, JNK, and p38 (Figure 6B–D). However, these effects were dose-dependently suppressed by the DPDS treatment. Altogether, the NFκB and MAPK pathways could be down-regulated by treatment with DPDS, thereby contributing to the decrease in intracellular TNF-α, IL-6 and IL-1β levels.

## 3. Discussion

One of the pathogenic mechanisms of DN is the excessive production of ROS and the occurrence of OS in the kidney [29,30]. Moreover, ROS are also involved in various biological processes related to DN, such as cell proliferation, apoptosis and ECM deposition [31]. Increasing evidence has shown that antioxidant therapy is beneficial in preventing and treating diabetic kidney disease [32,33,34]. Therefore, the application of antioxidants to prevent and relieve DN has received increasing attention. DPDS, a simple and stable diaryl diselenide, has received widespread attention due to its potent antioxidant and anti-hyperglycemic effects. The results presented here indicate that DPDS treatment increased the viability of t-BHP-treated HBZY-1 cells and exerted a profound protective effect on t-BHP-induced cytotoxicity. Therefore, it is necessary to explore its protective mechanism.

The excessive production of ROS is a direct consequence of hyperglycemia [35], which can result in apoptosis of proximal tubule epithelial cells and mesangial cells of the kidney, and the depletion of podocytes, thereby promoting the progression of DN [36,37]. In addition, GSH, SOD, and CAT are important components of the antioxidant defense system, which can work synergistically to clear excess ROS in the body [38]. SOD plays a key role in maintaining the body’s oxidation and antioxidant balance by catalyzing the dismutation of superoxide anions [39,40]. MDA is usually used as an indicator of the occurrence of OS and is one of the most important products of membrane lipid peroxidation. The GSH system acts as a key endogenous factor to protect against OS, and plays a major role in protecting against free radicals and ROS insults in most mammalian cells [41]. Therefore, we next measured these indicators related to oxidative stress in HBZY-1 cells to ascertain whether DPDS protects HBZY-1 cells against t-BHP-induced cytotoxicity via suppressing OS. The data showed that t-BHP exposure enhanced ROS level and MDA content, and reduced GSH level and SOD activity in HBZY-1 cells. However, after exposure to DPDS, these changes were significantly reversed, indicating that DPDS is able to maintain the redox homeostasis in t-BHP-treated HBZY-1 cells and plays a crucial antioxidant role in this model.

Therefore, the above results motivate us to further explore the underlying action mechanisms of DPDS in improving OS. The Nrf2/Keap1 signaling pathway represents one of the most important endogenous antioxidant pathways [42]. Nrf2 and its target genes are crucial components of endogenous anti-oxidative systems. The Nrf2 signaling pathway is able to attenuate ROS and regulate redox homeostasis under oxidative stress, so it has an important defensive effect on DN [43]. In fact, Nrf2 is highly sensitive to OS. Under normal circumstances, Nrf2 binds to its inhibitory protein Keap1 in the cytoplasm and thus lacks activity. Once stimulated by OS or other factors, Nrf2 dissociates from Keap1 and subsequently enters the cell nucleus to bind to specific DNA fragments, and induces the transcription and translation of downstream genes, such as SOD, GCLC, NQO1, and HO-1, for antioxidation and detoxication [44]. Accordingly, Nrf2 is currently considered as a potential and effective therapeutic target for preventing and treating DN. In order to ascertain whether DPDS can regulate the Nrf2 pathway in t-BHP-treated HBZY-1 cells, the protein levels and mRNA expression of the Nrf2-related signaling molecules were detected. The t-BHP treatment caused significant decreases in the protein expression of Nrf2, NQO1, HO-1, and GCLC, as well as significant decreases in the gene levels of *Nrf2*, *NQO1*, *HO-1*, *GCLC*, and *GPX1*. In contrast, the gene level of *Keap1* was elevated after t-BHP treatment in HBZY-1 cells. However, the DPDS treatment mitigated these effects to a certain extent. These results indicate that DPDS exerts powerful antioxidant activity by activating Nrf2 signaling, which may contribute to the maintenance of long-term redox homeostasis in cells. In line with this, it has been reported that DPDS can improve OS via activating the Nrf2 system in the liver or macrophage cells [45,46]. As far as we know, our study is the first to show that DPDS can activate the Nrf2/Keap1 pathway in a cell model of DN, indicating that the activation of the Nrf2/Keap1 pathway is involved in the protective effects of DPDs on t-BHP-induced cytotoxicity in HBZY-1 cells. Furthermore, the in vitro results are consistent with our previous in vivo findings regarding the antioxidant effects of DPDS [24], which further confirmed the ameliorative effect of DPDS on DN. Nevertheless, the mechanisms underlying the activation of the Nrf2 pathway by DPDS remain elusive and merit further investigation. Moreover, ferroptosis promotes the development of DN by affecting the functions of renal podocytes, mesangial cells and renal tubular cells, which are closely related to the occurrence and development of DN [47]. In addition, studies have shown that the trace element selenium plays an important role in regulating ferroptosis [48]. Therefore, the improvement of DN by DPDS may be related to the regulation of ferroptosis, which was not covered in our study. Hence, further studies on the effects of DPDS on ferroptosis are worthwhile in follow-up programs for DN.

In addition, the activation of the innate immune system and inflammatory response play crucial roles in the pathogenesis and development of DN [12]. Many studies have found that various inflammatory cytokines are evidently increased in the early stage of DN, contributing to the development of DN [49]. IL-6, a cytokine of the chemokine family, is connected to structural and functional abnormalities of diabetic kidney, including abnormal glomerular endothelial permeability, mesangial cell expansion, and elevated fibronectin expression [50,51]. Additionally, IL-1β is closely related to abnormal hemodynamic changes in the glomerulus, which can increase the synthesis of glomerular type IV collagen, and eventually cause the expansion of the glomerular matrix. In addition, TNF-α can enhance the generation of free radicals in glomerular mesangial cells, which directly leads to kidney damage [12,52]. In the current study, the LPS treatment caused an inflammatory response in HBZY-1 cells, as demonstrated by significantly elevated intracellular contents and gene levels of *IL-1β*, *IL-6*, and *TNF-α*. However, the DPDS treatment effectively alleviated LPS-induced inflammatory response, consistent with early studies showing the anti-inflammatory activity of DPDS [17].

Next, we explored the possible molecular mechanism by which DPDS improves inflammation. NFκB, a transcription factor found in B lymphocyte precursor cells [53], is closely related to the production of various inflammatory cytokines and the regulation of inflammatory response in the body [54], and participates in multiple biological processes [53,54]. In mouse models of DN, researchers have found that NFκB was significantly activated in renal proximal tubule cells [55,56] and renal cortex tissue of the kidney [57]. In addition, proteinuria can also stimulate the activation of renal NFκB, which is one of the important pro-inflammatory stimuli in renal tubular cells [55]. In this study, DPDS was found to inhibit LPS-induced NFκB phosphorylation in HBZY-1 cells, indicating that the NFκB signaling pathway is involved in the inhibition of inflammation by DPDS. Moreover, the MAPK signaling pathway is involved in the activation of the NFκB pathway. MAPK is composed of four subtypes including ERK1/2, p38, JNK, and ERK5, and can be activated by hormones, neurotransmitters, cytokines, cell adhesion and cell stress, etc. [58]. The main feature of MAPK activation is the increased phosphorylation of ERK, P38, and JNK [59]. The MAPK signaling pathway regulates various inflammatory responses related to renal fibrosis. Intriguingly, it has been shown that inhibiting the MAPK pathway can attenuate DN [60,61,62]. On the other hand, the MAPK pathway is a classical pathway that can initiate the production of inflammatory mediators and NFκB activation indirectly or directly. Accordingly, we further examined the influence of DPDS on the MAPK signaling pathway. DPDS treatment for 24 h reduced the phosphorylation of JNK and ERK1/2, and may thus inhibit LPS-induced NFκB phosphorylation and decrease the contents of intracellular IL-1β, IL-6, and TNF-α, eventually exerting an anti-inflammatory action. Taken together, the anti-inflammatory activity of DPDS in HBZY-1 cells is attributed, at least in part, to the suppression of NF-κB and MAPK signaling pathways. Notably, in the inflammatory cell model, we did not examine whether DPDS also im-proved LPS-induced cell injury after the use of MAPK and NFκB signaling pathway inhib-itors, which is one of the limitations of this study. Nevertheless, the relationship between the improvement of inflammatory response by DPDS and the MAPK and NFκB pathways needs to be further clarified in the future.

Our study also had some limitations. Firstly, we did not use a model of HAZY-1 cell formation induced by high glucose concentrations to study the effects of DPDS on oxida-tive stress and the inflammatory response, which reduced the persuasiveness of our re-search. Secondly, we used only one cell line in our study and did not investigate the effects of DPDS on other cell lines in the kidney or on cells directly derived from patients. Although DPDS has been found to have a variety of pharmacological effects, it is still a long way from clinical application. It would be more significant if relevant data were available from the cells of patients. Thirdly, this study only investigated the preventive effects of DPDS and did not elucidate the therapeutic effects of DPDS in model-forming cells. Lastly, inhibitors were not used for validation in studies of the effects of DPDS on the MAPK and NFκB signaling pathways, and appropriate positive control agents were not used. Nevertheless, the further investigation of DPDS is very desirable, because it has many known pharmacological effects and unknown pharmacological effects that deserve further study. However, further research is needed to better understand the protective mechanism of DPDS against DN.

## 4. Materials and Methods

### 4.1. Reagents and Antibodies

Diphenyl diselenide (180629, C_12_H_10_Se_2_, MW: 312.13, purity: ≥ 98%), t-BHP (458139), LPS (SMB00610), 100× penicillin, and streptomycin (TMS-AB2) were obtained from Sigma-Aldrich (St. Louis, MO, USA). The cell culture medium (11965084, Dulbecco’s Modified Eagle’s Medium, DMEM), and serum (10099141C, fetal bovine serum; FBS) were purchased from Gibco (MD, USA). Kits for detecting GSH (A006-2-1), MDA (A003-4-1), and SOD (A001-3-2) levels were provided by Nanjing Jiancheng Bioengineering Institute (Nanjing, China). ELISA kits for measuring IL-6 (DG94490Q), IL-1β (DG94484Q), and TNF-α (DG20065D) contents were obtained from Beijing Dongge Boye Biotechnology Co. Ltd. (Beijing, China). A ROS (S0033S) detection kit was obtained from Beyotime Institute of Biotechnology (Beijng, China). Antibodies against Keap1 (8047S), NQO1 (3187S), NFκB (8242S), phospho-NFκB (3033S, p-NFκB), JNK (9252S), phospho-JNK (9255S, p-JNK), ERK1/2 (4695S), phospho-ERK1/2 (4370S, p-ERK1/2), p38 (8690S), and phospho-p38 (4511S, p-p38) were purchased from Cell Signaling Technology (Danvers, MA, USA). Antibodies against Nrf2 (ab137550), GCLC (ab190685), and HO-1 (ab189491) were acquired from Abcam (Cambridge, Cambridgeshire, UK). Polyvinylidene difluoride (IPFL00010, PVDF) membranes (Millipore, CA, USA), a bicinchoninic acid (p1511-2, BCA) protein kit, protease and phosphatase inhibitors (P1261), enhanced chemiluminescence (P1050-3, ECL) reagent, anti-β-actin (C1828-100), and the horseradish peroxidase (HRP)-labeled goat anti-rabbit (C1309-1) and mouse (C1308-1) IgG were purchased from Applygen Technologies (Beijing, China). TRIzol (15596018) reagent was provided by Invitrogen (Carlsbad, CA, USA). Transcript An All-in-One First-Strand cDNA Synthesis SuperMix (AE341-02) and a TransStart Tip Green qPCR SuperMix (AQ111-01) were obtained from TransGen Biotech (Beijing, China).

### 4.2. Cell Culture

HBZY-1 cells were purchased from the China Center for Type Culture Collection (Wuhan, China), and maintained in DMEM supplemented with 10% FBS, 0.1 g/L streptomycin, and 100 U/mL penicillin, and in a CO_2_ incubator (humidified, 37 °C, 5% CO_2_).

### 4.3. Cell Viability Assay

A CCK8 kit (CK04) was employed to measure the cell viability. Briefly, the cells were exposed to indicated concentrations of t-BHP and/or indicated concentrations of DPDS for 24 h. After that, 10 μL of CCK8 (Dojindo, Japan) was added to each well, followed by a further incubation for 2 h. Finally, a multifunctional microplate reader (BioTek, Synergy2, Winooski, VT, USA) was employed to measure the OD value at 450 nm of each well.

### 4.4. Assessment of ROS Levels

The production of intracellular ROS in HBZY-1 cells was evaluated with a ROS Assay Kit of DCFH-DA. HBZY-1 cells were exposed to t-BHP (400 μM) alone or in combination with various concentrations of DPDS for 24 h. After treatment, the cells were exposed to DCFH-DA in the dark for 30 min. After rinsing three times with PBS, a multifunctional microplate reader was employed to detect the fluorescence intensity of each well (Excitation-480 nm, Emission-525 nm).

### 4.5. Measurement of Intracellular Oxidative Stress Parameters

HBZY-1 cells were exposed to t-BHP (400 μM) alone or in combination with DPDS (10, 25 or 50 μM) for 24 h. After rinsing three times with PBS, the collected cells were homogenized and centrifuged (12,000 rpm, 10 min, 4 °C), and then the supernatant was used to evaluate the levels of MDA, GSH, and SOD activity. These parameters were quantified using the corresponding kits, following the vendor’s instructions and normalized to protein concentrations.

### 4.6. Enzyme-Linked Immunosorbent Assay (ELISA)

HBZY-1 cells were exposed to LPS (1 μg/mL) alone or in combination with DPDS (10, 25 or 50 μM) for 24 h. After rinsing three times with PBS, the collected cells were homogenized and centrifuged (12,000 rpm, 10 min, 4 °C), and then the supernatant was used to detect the levels of TNF-α, IL-6, and IL-1β. These factors were evaluated by the corresponding ELISA kits, following the vendor’s instructions and normalized to protein concentrations.

### 4.7. Western Blot

HBZY-1 cells were exposed to t-BHP (400 μM) or LPS (1 μg/mL) alone or in combination with DPDS (10, 25 or 50 μM) for 24 h. The cells were harvested after complete incubation and lysed in RIPA lysis buffer (C1053-1) containing protease inhibitor cocktail. After centrifugation, the supernatant was collected and quantified for protein concentrations using a BCA kit. The subsequent SDS-PAGE gel electrophoresis and immunoblotting were the same as previously described [63].

### 4.8. Gene Expression Analysis

HBZY-1 cells were exposed to indicated concentrations of agents for 24 h. Then, the cells were harvested and total RNA was isolated using TRIzol reagent, according to the vendor’s instructions. The method of cDNA synthesis and quantitative RT-PCR were the same as previously described [63,64]. The results were expressed as relative fold changes and quantitatively analyzed by the delta–delta Ct (2-ΔΔCt) method. Part of the primer sequences (Invitrogen, Beijing, China) used were: *TNF-α* 5′-TTGAACCAAGCATCACGGGT-3′ (forward) and 5′-TCGCCAGTCCTAACATCAGC-3′ (reverse), *IL-6* 5′-AGCGATGATGCACTGTCAGA-3′ (forward) and 5′-GGAACTCCAGAAGACCAGAGC-3′ (reverse), *IL-1β* 5′-GATGACGAGCGACTGTTCCA-3′ (forward) and 5′-TGGTAACCGCTCAGGTGTTG-3′ (reverse). The primer sequences for other genes were those described previously [64].

### 4.9. Statistical Analysis

All the values are presented as means ± SEM. Data were analyzed using one-way ANOVA with Bonferroni corrections. Values of *p* < 0.05 were considered to be statistically significant.

## 5. Conclusions

**Figure 7:**
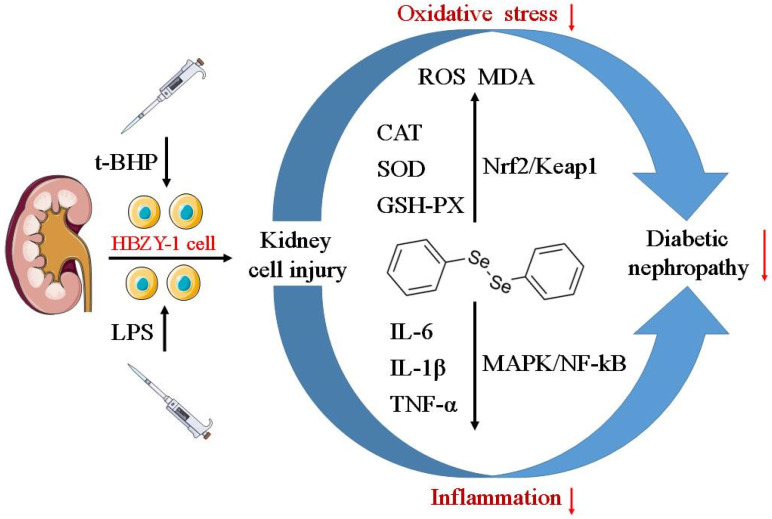
Schematic representation indicating the potential mechanism by which diphenyl diselenide exerts protective effects on t-BHP-induced oxidative stress and LPS-induced inflammation in HBZY-1 cells.

In conclusion, the protective effects of DPDS on t-BHP-induced OS and LPS-induced inflammatory responses were, for the first time, examined in our study. The activation of the Nrf2/Keap1 signaling pathway is involved in the antioxidant mechanism of DPDS. Moreover, the suppression of NFκB and MAPK signaling pathways is involved in the anti-inflammatory mechanism of DPDS (as shown in Figure 7). DPDS may represent a potential candidate for the prevention and treatment of DN. However, the present study is a preliminary in vitro study, and further in vivo investigation is warranted to confirm the protective effect of DPDS on DN.

## Data Availability

Data sharing is not applicable to this article.
